# Supplementary material for: The relationship between wasting and stunting in Cambodian children: Secondary analysis of longitudinal data of children below 24 months of age followed up until the age of 59 months
Source: PLoS One. 2021 Nov 18;16(11):e0259765. doi: 10.1371/journal.pone.0259765 (PMC8601787; doi:10.1371/journal.pone.0259765)
Supplement: S1 Fig — WHZ change and MUAC change at visit 0 to follow up visit 6. (PDF) [file pone.0259765.s001.pdf]

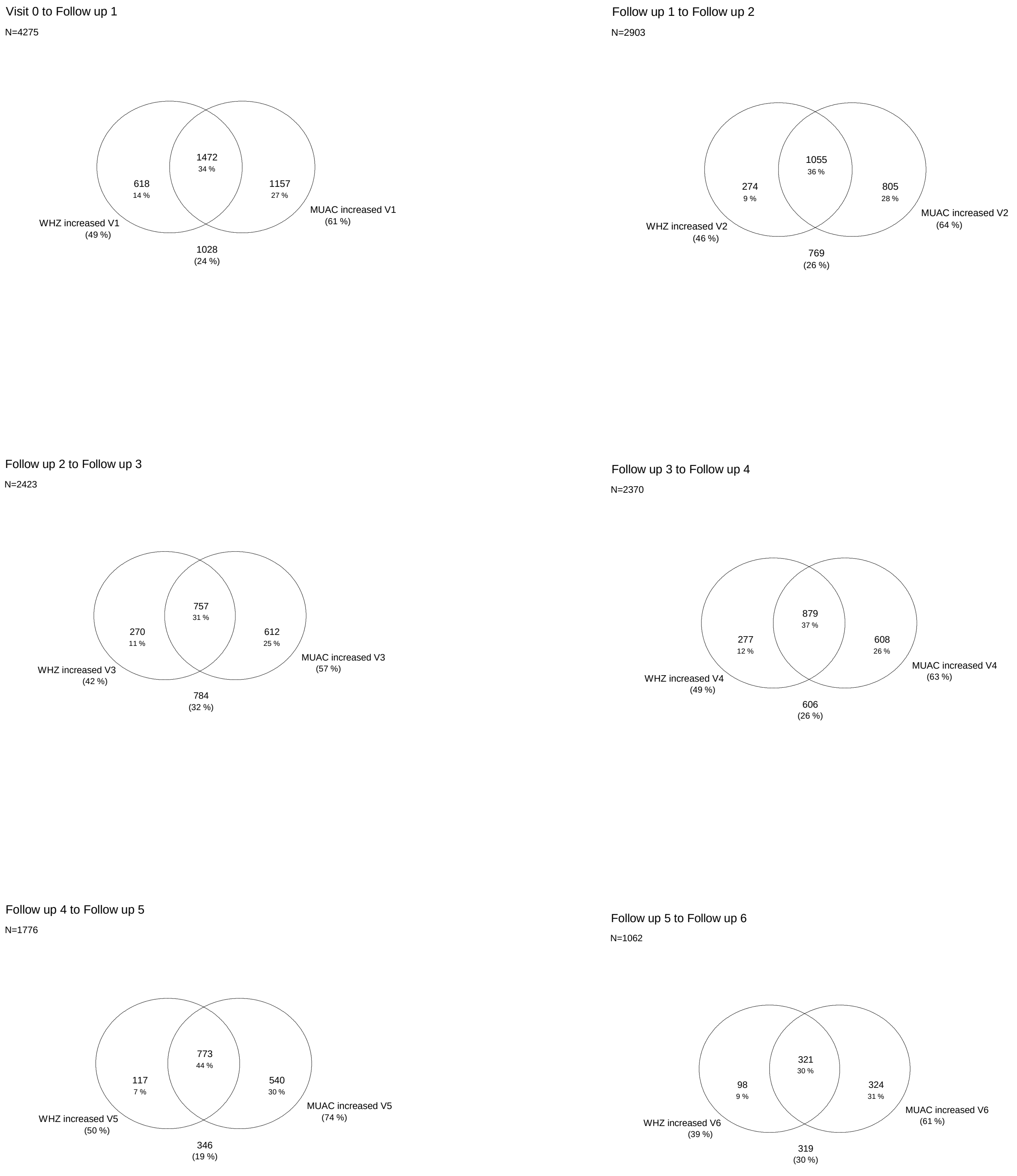

Supplementary figure 1 : Overlap between weight-for-height Z-score and mid-Upper Arm Circumference change categories during the different periods
