# Supplementary material for: The relationship between wasting and stunting in Cambodian children: Secondary analysis of longitudinal data of children below 24 months of age followed up until the age of 59 months
Source: PLoS One. 2021 Nov 18;16(11):e0259765. doi: 10.1371/journal.pone.0259765 (PMC8601787; doi:10.1371/journal.pone.0259765)
Supplement: S1 Table — HAZ change from visit 0 to follow up visit 6. (DOCX) [file pone.0259765.s002.docx]

**Supplementary table 1: Factors associated with height-for-age Z-score (HAZ) change at the different follow-up visits**

|  | Univariate analysis^1^ | | | Multivariate analysis^1^ | | |
| --- | --- | --- | --- | --- | --- | --- |
| Variable | Coef^2^ | (95%CI^3^) | p-value | Coef^2^ | (95%CI^3^) | p-value |
| *HAZ^4^ change: V0^5^ to FV1^6^ (n=4264)* |  |  |  |  |  |  |
| **Low MUAC^8^ at V0** |  |  |  |  |  |  |
| No: MUAC≥ 12.5 cm/ Yes: MUAC<12.5 cm | 0.00 | (-0.05; 0.06) | 0.896 | 0.18 | (0.12; 0.25) | <0.001 |
| MUAC change V0 to FV1 (cm) | 0.07 | (0.05; 0.09) | <0.001 | 0.13 | (0.11; 0.16) | <0.001 |
| **Stunted at V0** |  |  |  |  |  |  |
| No Z-score ≥-2/Yes: Z-score <-2 | -0.51 | (-0.56; -0.45) | <0.001 | -0.49 | (-0.55; -0.44) | <0.001 |
| **Sex** |  |  |  |  |  |  |
| Boys/Girls | 0.01 | (-0.04; 0.05) | 0.709 | -0.06 | (-0.10; -0.01) | 0.013 |
| **Age at VO (months)** |  |  |  |  |  |  |
| 0-5/12-23 | -0.19 | (-0.24; -0.14) | <0.001 | -0.15 | (-0.21; -0.09) | <0.001 |
| 6-11/12-23 | -0.20 | (-0.26; -0.14) | <0.001 | -0.12 | (-0.18; -0.06) | <0.001 |
| **Toilet type at V0** |  |  |  |  |  |  |
| Improved /Unimproved | -0.05 | (-0.10; -0.00) | 0.032 | -0.04 | (-0.01; 0.19) | 0.020 |
| *HAZ change: FV1 to FV2 (n=2852)* |  |  |  |  |  |  |
| **Wasted at V0** |  |  |  |  |  |  |
| No Z-score ≥-2 /Yes: Z-score <-2 | -0.10 | (-0.16; -0.03) | 0.003 | 0.10 | (0.04; 0.17) | 0.002 |
| Change in WHZ: VO to FV1 | 0.12 | (0.10; 0.14) | <0.001 | 0.13 | (0.10; 0.15) | <0.001 |
| MUAC change FV1 to FV2 (cm) | 0.10 | (0.07; 0.13) | <0.001 | 0.10 | (0.07; 0.13) | <0.001 |
| **Stunted at V1** |  |  |  |  |  |  |
| No Z-score ≥-2 /Yes: Z-score <-2 | -0.32 | (-0.36; -0.27) | <0.001 | -0.30 | (-0.34; -0.25) | <0.001 |
| **Age at VO (months)** |  |  |  |  |  |  |
| 0-5/12-23 | -0.20 | (-0.25; -0.15) | <0.001 | -0.11 | (-0.15; -0.06) | <0.001 |
| 6-11/12-23 | -0.04 | (-0.09; 0.02) | 0.165 | 0.01 | (-0.04; 0.07) | 0.614 |
| **Water source type at V0** |  |  |  |  |  |  |
| Improved/Unimproved | -0.08 | (-0.13; -0.02) | 0.004 | -0.03 | (-0.06; 0.11) | 0.068 |
| HAZ change: FV2 to FV3 (n=2157) |  |  |  |  |  |  |
| **Wasted at FV1** |  |  |  |  |  |  |
| No Z-score ≥-2/Yes: Z-score <-2 | -0.05 | (-0.12; 0.02) | 0.191 | 0.13 | (0.06; 0.20) | <0.001 |
| Change in WHZ: FV1 to FV2 | 0.21 | (0.18; 0.24) | <0.001 | 0.19 | (0.16; 0.22) | <0.001 |
| MUAC change FV2 to FV3 (cm) | 0.11 | (0.08; 0.14) | <0.001 | 0.09 | (0.06; 0.12) | <0.001 |
| **Stunted at FV2** |  |  |  |  |  |  |
| No Z-score ≥-2/Yes: Z-score <-2 | -0.26 | (-0.31; -0.21) | <0.001 | -0.22 | (-0.26; -0.18) | <0.001 |
| **Age at VO (months)** |  |  |  |  |  |  |
| 0-5/12-23 | -0.27 | (-0.32; -0.23) | <0.001 | -0.18 | (-0.22; -0.13) | <0.001 |
| 6-11/12-23 | -0.13 | (-0.18; -0.09) | <0.001 | -0.09 | (-0.15; -0.04) | <0.001 |
| **Water source type at V0** |  |  |  |  |  |  |
| Improved/ Unimproved | 0.04 | (-0.01; 0.09) | 0.094 | 0.05 | (0.01; 0.10) | 0.029 |
| *HAZ change: FV3 to FV4 (n=2000)* |  |  |  |  |  |  |
| **Wasted at FV2** |  |  |  |  |  |  |
| No Z-score ≥-2/Yes: Z-score <-2 | 0.03 | (-0.03; 0.10) | 0.318 | 0.13 | (0.07; 0.20) | <0.001 |
| Change in WHZ: FV2 to FV3 | 0.13 | (0.10; 0.16) | <0.001 | 0.14 | (0.11; 0.17) | <0.001 |
| MUAC change FV3 to FV4 (cm) | 0.07 | (0.04; 0.10) | <0.001 | 0.07 | (0.05; 0.10) | <0.001 |
| **Stunted** **at V3** |  |  |  |  |  |  |
| No Z-score ≥-2 /Yes: Z-score <-2 | -0.20 | (-0.22; -0.14) | <0.001 | -0.19 | (-0.23; -0.15) | <0.001 |
| **Age at VO (months)** |  |  |  |  |  |  |
| 0-5/12-23 | -0.18 | (-0.32; -0.23) | <0.001 | -0.13 | (-0.17; -0.09) | <0.001 |
| 6-11/12-23 | -0.04 | (-0.09; 0.00) | 0.60 | -0.04 | (-0.09; -0.00) | 0.050 |
| **Sex** |  |  |  |  |  |  |
| Boys /Girls | 0.06 | (0.02; 0.10) | 0.002 | 0.05 | (0.01; 0.08) | 0.009 |
| **Mother years of formal education** |  |  |  |  |  |  |
| 7-9 years/0-6 years | 0.03 | (-0.02; 0.08) | 0.205 | 0.04 | (-0.01; 0.08) | 0.103 |
| ≥710 years/0-6 years | 0.03 | (-0.03; 0.09) | 0.289 | 0.06 | (-0.0; 0.11) | 0.051 |
| **Toilet type at V0** |  |  |  |  |  |  |
| Improved /Unimproved | 0.01 | (-0.03; 0.05) | 0.751 | 0.03 | (-0.01; 0.07) | 0.115 |
| *HAZ change: FV4 to FV5 (n=1453)* |  |  |  |  |  |  |
| **Wasted at FV3** |  |  |  |  |  |  |
| No Z-score ≥-2/Yes: Z-score <-2 | -0.05 | (-0.14; 0.04) | 0.276 | 0.05 | (-0.04; 0.13) | 0.309 |
| Change in WHZ: FV3 to FV4 | 0.10 | (0.06; 0.15) | <0.001 | 0.10 | (0.05; 0.14) | <0.001 |
| MUAC change FV4 to FV5 (cm) | 0.11 | (0.08; 0.15) | <0.001 | 0.14 | (0.10; 0.17) | <0.001 |
| **Stunted at V4** |  |  |  |  |  |  |
| No Z-score ≥-2/Yes: Z-score <-2 | -0.21 | (-0.26; -0.15) | <0.001 | -0.18 | (-0.24; -0.13) | <0.001 |
| **Age at V0 (months)** |  |  |  |  |  |  |
| 0-5/12-23 | -0.10 | (-0.16; -0.04) | 0.001 | -0.14 | (-0.20; -0.08) | <0.001 |
| 6-11/12-23 | -0.03 | (-0.09; 0.03) | 0.358 | -0.07 | (-0.13; -0.00) | 0.048 |
| **Sex** |  |  |  |  |  |  |
| Boys/Girls | 0.10 | (0.04; 0.15) | <0.001 | 0.08 | (0.03; 0.14) | 0.001 |
| *HAZ change: FV5 to FV6 (n=842)* |  |  |  |  |  |  |
| **Wasted at FV4** |  |  |  |  |  |  |
| No Z-score ≥-2/Yes: Z-score <-2 | 0.09 | (0.02; 0.17) | 0.018 | 0.10 | (0.02; 0.17) | 0.011 |
| Change in WHZ: FV4 to FV5 | 0.22 | (0.18; 0.26) | <0.001 | 0.22 | (0.18; 0.28) | <0.001 |
| MUAC change FV5 to FV6 (cm) | 0.03 | (-0.01; 0.06) | 0.146 | 0.05 | (0.01; 0.08) | 0.011 |
| **Stunted at V0** |  |  |  |  |  |  |
| No Z-score ≥-2 /Yes: Z-score <-2 | -0.21 | (-0.26; -0.15) | <0.001 | -0.10 | (-0.15; -0.05) | <0.001 |
| **Age at VO (months)** |  |  |  |  |  |  |
| 0-5/12-23 | -0.08 | (-0.14; -0.02) | 0.007 | -0.10 | (-0.15; -0.06) | <0.001 |
| 6-11/12-23 | -0.09 | (-0.14; -0.03) | 0.003 | -0.11 | (-0.17; -0.06) | <0.001 |
| **Sex** |  |  |  |  |  |  |
| Boys/Girls | 0.06 | (0.01; 0.11) | 0.010 | 0.007 | (0.03; 0.12) | 0.002 |

^1^Computed using multilevel regression analysis test (stata mixed command); ^2^Coef=coefficient; ^3^CI=confidence Interval;  ^4^HAZ= Length/height-for-age Z-score (2006 WHO reference curves); stunted if HAZ<-2 ; ^5^V0= recruitment into the cohort visit;  ^6^FV= follow up visit; ^7^WHZ= weight-for-length/height Z-score (2006 WHO reference curves); wasted=yes if WHZ<-2; ^8^MUAC=Mid-Upper Arm Circumference
