# Supplementary material for: The relationship between wasting and stunting in Cambodian children: Secondary analysis of longitudinal data of children below 24 months of age followed up until the age of 59 months
Source: PLoS One. 2021 Nov 18;16(11):e0259765. doi: 10.1371/journal.pone.0259765 (PMC8601787; doi:10.1371/journal.pone.0259765)
Supplement: S2 Table — Change in wasting and stunting parameters from visit 0 to follow up visit 6. (DOCX) [file pone.0259765.s003.docx]

**Supplementary table 2: Association between stunting, wasting parameters and occurrence of accelerated linear growth at the different follow up visits.**

| **Predictors** |  |  | **Univariate analysis^1^** | | | | **Multivariate analysis^1^** | | | |
| --- | --- | --- | --- | --- | --- | --- | --- | --- | --- | --- |
|  | **n** | **% ALG^2^** | **OR^3^** | **95%CI^4^** | | **p-value** | **AOR^5^** | **95%CI^4^** | | **p-value** |
|  |  |  |  | **LL** | **UL** |  |  | **LL** | **UL** |  |
| **Follow up visit 1 (n=4342)** |  |  |  |  |  |  |  |  |  |  |
| Wasting at V0^6^ |  |  |  |  |  |  |  |  |  |  |
| YES: WHZ^7^<-2 Z-score | 513 | 5.16 | 0.75 | 0.50 | 1.13 | 0.169 | 1.56 | 0.96 | 2.53 | 0.070 |
| NO: WHZ≥-2-score | 3829 | 6.82 | Base |  |  |  | Base |  |  |  |
| MUAC^8^ group at V0 |  |  |  |  |  |  |  |  |  |  |
| <12.5 cm | 922 | 10.64 | 2.19 | 1.70 | 2.83 | <0.001 | 1.92 | 1.33 | 2.78 | <0.001 |
| ≥12.5 cm | 3377 | 5.26 | Base |  |  |  | Base |  |  |  |
| Stunting at V0 |  |  |  |  |  |  |  |  |  |  |
| YES: HAZ^9^<-2-score | 871 | 14.15 | 3.89 | 3.04 | 4.97 | <0.001 | 4.30 | 3.23 | 5.73 | <0.001 |
| No: HAZ≥-2 Z-score | 3472 | 4.51 | Base |  |  |  | Base |  |  |  |
| WHZ change V0 to FV^10^1 : | 4342 | 6.63 | 0.54 | 0.48 | 0.61 | <0.001 | 0.50 | 0.43 | 0.58 | <0.001 |
| MUAC change V0 to FV1 | 4274 | 6.63 | 1.666 | 1.51 | 1.81 | <0.001 | 2.01 | 1.76 | 2.30 | <0.001 |
| **Follow up visit 2 (n=2928)** |  |  |  |  |  |  |  |  |  |  |
| Wasting at V0 |  |  |  |  |  |  |  |  |  |  |
| YES: WHZ<-2 Z-score | 259 | 3.93 | 1.23 | 0.73 | 2.06 | 0.431 | 0.88 | 0.47 | 1.65 | 0.681 |
| NO:WHZ≥-2-score | 2643 | 3.65 | Base |  |  |  | Base |  |  |  |
| MUAC group at FV1 |  |  |  |  |  |  |  |  |  |  |
| <12.5 cm | 314 | 4.16 | 1.21 | 0.71 | 2.08 | 0.487 | 0.61 | 0.3 | 116 | 0.132 |
| ≥12.5 cm | 2603 | 3.92 | Base |  |  |  | Base |  |  |  |
| Stunting at FV1 |  |  |  |  |  |  |  |  |  |  |
| YES:HAZ<-2-score | 722 | 6.86 | 3.45 | 2.38 | 5.00 | <0.001 | 4.97 | 3.31 | 7.47 | <0.001 |
| No: HAZ≥-2 Z-score | 2206 | 2.47 | Base |  |  |  | Base |  |  |  |
| WHZ change V0 to FV1 : | 2876 | 3.68 | 1.24 | 1.02 | 1.51 | 0.030 | 1.29 | 1.04 | 1.59 | 0.019 |
| MUAC change FV1 to FV2 | 2842 | 3.99 | 1.71 | 1.33 | 2.21 | <0.001 | 1.96 | 1.50 | 2.58 | <0.001 |
| **Follow up visit 3 (n=2157)** |  |  |  |  |  |  |  |  |  |  |
| Wasting at FV1 |  |  |  |  |  |  |  |  |  |  |
| YES:WHZ≥-2-score | 189 | 1.79 | 0.89 | 0.34 | 2.25 | 0.805 | 0.37 | 0.13 | 1.06 | 0.063 |
| No: WHZ<-2 Z-score | 1968 | 2.76 | Base |  |  |  | Base |  |  |  |
| MUAC group at FV2 |  |  |  |  |  |  |  |  |  |  |
| <12.5 cm | 150 | 2.04 | 0.93 | 0.33 | 2.91 | 0.892 | 0.78 | 0.25 | 2.41 | 0.663 |
| ≥12.5 cm | 2007 | 2.73 | Base |  |  |  | Base |  |  |  |
| Stunting at FV2 |  |  |  |  |  |  |  |  |  |  |
| YES:HAZ<-2-score | 626 | 4.92 | 3.53 | 2.13 | 5.86 | <0.001 | 3.71 | 2.18 | 6.31 | <0.001 |
| No: HAZ≥-2 Z-score | 1531 | 1.61 | Base |  |  |  | Base |  |  |  |
| WHZ change FV1 to FV2: | 2157 | 2.67 | 2.23 | 1.64 | 3.03 | <0.001 | 2.38 | 1.70 | 3.31 | <0.001 |
| MUAC change FV2 to FV3 | 2157 | 2.67 | 1.06 | 0.73 | 1.53 | 0.774 | 2.28 | 1.62 | 3.20 | <0.001 |
| **Follow up visit 4 (n=2010)** |  |  |  |  |  |  |  |  |  |  |
| Wasting at FV2 |  |  |  |  |  |  |  |  |  |  |
| YES:WHZ≥-2-score | 186 | 1.52 | 0.71 | 0.22 | 2.31 | 0.564 | 0.30 | 0.08 | 1.10 | 0.069 |
| No: WHZ<-2 Z-score | 1824 | 2.03 | Base |  |  |  | Base |  |  |  |
| MUAC group at FV3 |  |  |  |  |  |  |  |  |  |  |
| <12.5 cm | 110 | 3.41 | 1.87 | 0.65 | 5.40 | 0.248 | 1.16 | 0.34 | 3.88 | 0.815 |
| ≥12.5 cm | 1900 | 1.88 | Base |  |  |  | Base |  |  |  |
| Stunting at FV3 |  |  |  |  |  |  |  |  |  |  |
| YES:HAZ<-2-score | 642 | 3.21 | 3.23 | 1.73 | 6.01 | <0.001 | 4.37 | 2.20 | 8.69 | <0.001 |
| No: HAZ≥-2 Z-score | 1368 | 1.29 | Base |  |  |  | Bas |  |  |  |
| WHZ change FV2 to FV1: | 2010 | 1.98 | 1.97 | 1.35 | 2.89 | <0.001 | 2.60 | 1.74 | 3.90 | <0.001 |
| MUAC change FV3 to FV1 | 2010 | 1.98 | 1.80 | 1.23 | 2.63 | 0.002 | 1.73 | 1.18 | 2.54 | <0.001 |
| **Follow up visit 5 (n=1453)** |  |  |  |  |  |  |  |  |  |  |
| Wasting at FV3 |  |  |  |  |  |  |  |  |  |  |
| YES:WHZ<-2-score | 147 | 1.74 | 0.91 | 0.32 | 2.62 | 0.869 | 0.59 | 0.19 | 1.86 | 0.367 |
| No: WHZ≥-2 Z-score | 1306 | 2.96 | Base |  |  |  | Base |  |  |  |
| MUAC group at FV4 |  |  |  |  |  |  |  |  |  |  |
| <12.5 cm | 58 | 0.72 | 0.58 | 0.08 | 4.32 | 0.595 | 0.60 | 0.07 | 5.17 | 0.641 |
| ≥12.5 cm | 1395 | 2.92 | Base |  |  |  | Base |  |  |  |
| Stunting at FV4 |  |  |  |  |  |  |  |  |  |  |
| YES:HAZ<-2-score | 511 | 2.90 | 1.46 | 0.79 | 2.70 | 0.231 | 1.44 | 0.76 | 2.71 | 0.261 |
| No: HAZ≥-2 Z-score | 942 | 2.74 | Base |  |  |  | Base |  |  |  |
| WHZ change FV3 to FV4: | 1453 | 2.98 | 2.06 | 1.27 | 3.36 | 0.004 | 2.17 | 1.30 | 3.61 | 0.003 |
| MUAC change FV4 to FV4 | 1453 | 2.98 | 2.15 | 1.51 | 3.07 | <0.001 | 2.31 | 1.61 | 2.31 | <0.001 |
| **Follow up visit 6 (n=1003)** |  |  |  |  |  |  |  |  |  |  |
| Wasting at FV4 |  |  |  |  |  |  |  |  |  |  |
| YES:WHZ<-2-score | 106 | 3.11 | 0.66 | 0.42 | 1.03 | 0.068 | 0.41 | 0.10 | 1.67 | 0.214 |
| No: WHZ≥-2 Z-score | 897 | 3.14 | Base |  |  |  | Base |  |  |  |
| MUAC group at FV5 |  |  |  |  |  |  |  |  |  |  |
| <12.5 cm | 14 | 7.69 | 1.88 | 0.24 | 14.67 | 0.547 | 1.31 | 0.10 | 16.31 | 0.834 |
| ≥12.5 cm | 989 | 3.06 | Base |  |  |  | Base |  |  |  |
| Stunting at FV5 |  |  |  |  |  |  |  |  |  |  |
| YES:HAZ<-2-score | 429 | 4.98 | 3.09 | 1.45 | 6.60 | 0.003 | 2.26 | 1.01 | 5.05 | 0.047 |
| No: HAZ≥-2 Z-score | 574 | 1.66 | Base |  |  |  | Base |  |  |  |
| WHZ change FV4 to FV5: | 1003 | 3.14 | 3.27 | 2.14 | 4.99 | <0.001 | 3.39 | 2.20 | 5.45 | <0.001 |
| MUAC change FV5 to FV6 | 1003 | 3.14 | 1.27 | 0.82 | 1.97 | 0.285 | 1.73 | 1.02 | 2.95 | 0.043 |

^1^Using multilevel logistic analysis (melogit STA command) with estimates analyses considering both the region and individual levels; ^2^ALG=Accelerated linear growth; ^2^ALG=accelerated linear growth as defined by the height-for-age change between the two assessments; ^3^OR= unadjusted region weighted odds ratio; ^4^CI=confidence interval; ^5^ AOR= adjusted region weighted odds ratio- the adjustment variables not presented in the table included age group, sex, mother's years of formal education, type of drinking water source and type of toilet facility of the household,^6^ WHZ=weight-for-height/length Z-score; ^7^V0= Visit 0 or recruitment visit; ^8^MUAC=Mid-Upper Arm Circumference; ^9^HAZ=Height/length-for-age Z-score; ^10^FV=follow up visit.
