# Supplementary material for: The relationship between wasting and stunting in Cambodian children: Secondary analysis of longitudinal data of children below 24 months of age followed up until the age of 59 months
Source: PLoS One. 2021 Nov 18;16(11):e0259765. doi: 10.1371/journal.pone.0259765 (PMC8601787; doi:10.1371/journal.pone.0259765)
Supplement: Attachment — Submitted filename: Review2_Wasting and Stunting.docx [file pone.0259765.s011.docx]

Dear editor,

dear authors,

thank you for the opportunity to re-review the paper again, which deals in general with a very important and interesting topic. Unfortunately, the authors did not deal with my major concerns from the first review. Most of the very helpful reviewers’ concerns (and I mean also the concerns from the other reviewers) were rejected by authors without integrating them in the paper. This is disappointing since the remarks would had improved the paper significantly.

There are still two major concerns that were (still) fully neglected by the authors although the reviewers mentioned several times. 1) statistical methods, 2) the paper structure/writing. Due to these ignored major concerns I have to recommend to reject the paper and did not consider for a further review. Let me explain this more detailed in the following paragraph:

1) Statistical methods

The authors write in lines 84-86 that children were surveyed every 3 to 4 months, so that it makes the impression the authors used panel data. The authors answered that they indeed did not perform panel data analysis. The authors’ argumentation that “the cross-sectional/open cohort type of the data collection applied in the original study. Also, the level of missings when trying to apply panel data analysis suggested that the obtained estimates would likely be biased.” is poor or did not fit with the authors data description in the source of data section. Panel analysis can also be conducted when the data were of unbalanced nature why the argumentation is not correct from my view. Additionally, which kind of study design/ analysis strategy was used? Did the authors estimate seemingly unrelated regressions? If so, why they did not mention in the Method section? Altogether, the methodology used in the paper remained still open and I have major concerns that the statistical methods were conducted properly. Altogether, the data and method section was written poor and did not sound convincing that the authors did, in fact, perform the proper statistical methods.

1) Paper structure/writing

The paper is too lengthy, which was already mentioned in review round 1. Especially the method and discussion parts were written poor. Although the reviewers mentioned that several times, the authors did not integrate and disagreed. This is a bit unprofessional kind of responding to a review. It was already mentioned by another reviewer that the research question should be mentioned in the introduction – the authors did not regard this remark. The method section mainly focused on STATA commands and did not explain the used methods in a professional fashion by using equations or, at least, correct explanations concerning the procedure. This was mentioned in my review from round 1. Both was missed in the paper, and not integrated although it was mentioned in review 1.

I recommended in my first review, how the authors could structure their discussion to improve the readability of the paper. They neglected completely! Arguing that PLOS ONE is an open format journal cannot be used as a good argument to ignore general remarks for paper improvements…
